# Clinical value of C-reactive protein to albumin ratio, aspartate aminotransferase, and platelet-to-lymphocyte ratio in predicting the severity of community-acquired pneumonia in children

**DOI:** 10.3389/fped.2025.1611792

**Published:** 2025-10-03

**Authors:** Bo Han, Xiang Li, Lu Zhang, Hao Zhang, Bo Wang

**Affiliations:** ^1^ Department of Clinical Laboratory, First Affiliated Hospital of Anhui Medical University, Anhui, China; ^2^ The People’s Hospital of Chi Zhou, Chizhou, China; ^3^ Faculty of Medicine, Macau University of Science and Technology, Macau, China; ^4^ The Second Affiliated Hospital of Anhui Medical University, Hefei, China

**Keywords:** community-acquired pneumonia, severe pneumonia, risk factors, inflammatory markers, C-reactive protein to albumin ratio

## Abstract

**Background/objectives:**

Community-Acquired Pneumonia (CAP) is a common acute and critical illness in pediatrics and one of the leading causes of death in hospitalized children worldwide. Inflammatory mechanisms play a significant role in pneumonia, and inflammatory markers have shown value in predicting severity in various diseases. This study aimed to explore the predictive value of key inflammatory markers for identifying the severity of pediatric CAP.

**Methods:**

A retrospective analysis was conducted on children with CAP, comparing inflammatory markers between mild and severe pneumonia groups. Markers analyzed included Platelet-to-Lymphocyte Ratio (PLR), Aspartate Aminotransferase (AST), and C-Reactive Protein to Albumin Ratio (CAR). Statistical analyses involved group comparisons using appropriate tests for continuous and categorical variables, multivariate logistic regression to identify independent risk factors, and receiver operating characteristic (ROC) curves to evaluate predictive performance (*p* < 0.05 considered significant).

**Results:**

A total of 303 children were included; 87 with severe pneumonia and 216 with mild pneumonia. Severe cases showed significantly higher levels of CAR (24.52 vs. 0.19 mg/L, *p* = 0.004), AST (37.67 vs. 7.85 U/L, *p* = 0.040), and PLR (162.83% vs. 126.55%, *p* = 0.042) compared to mild cases. Logistic regression confirmed CAR (OR = 1.052, 95% CI: 1.004–1.102, *p* = 0.004), AST (OR = 1.087, 95% CI: 1.064–1.111, *p* = 0.002), and PLR (OR = 1.046, 95% CI: 0.996–1.099, *p* = 0.033) as independent risk factors for severe CAP. ROC analysis showed AST had the highest discriminative power (AUC = 0.837, sensitivity = 78%, specificity = 85%), followed by CAR (AUC = 0.797) and PLR (AUC = 0.721).

**Conclusions:**

High levels of CAR, AST, and PLR are significantly associated with the presence of severe pneumonia in children with CAP and can serve as effective predictive indicators for identifying disease severity and guiding clinical assessment of disease progression.

## 1 Introduction

Community-Acquired Pneumonia (CAP) is a common respiratory disease caused by viruses, bacteria, chlamydia, and other microorganisms, with high prevalence in children and characteristics of high complication rates, severe illness, and rapid progression (1–4). It is one of the leading causes of death in children worldwide, significantly increasing the global disease burden and healthcare costs (5, 6). Early identification of severe pneumonia in children is crucial, but current severity grading and early warning scores for CAP patients have limitations such as high subjectivity, low sensitivity, and low specificity, making them less convenient for clinical use (7–9).

The pathogenesis of CAP involves multiple factors, including host defense mechanisms, microbial virulence, and environmental exposure (10–12). Treatment for CAP primarily involves antibiotic therapy, supportive care, and prevention of complications; however, due to antibiotic resistance, drug adverse reactions, and individual differences, treatment outcomes are not always ideal, with some patients progressing to requiring intensive care or mechanical ventilation, significantly increasing the risk of complications and adverse outcomes (13–15). Therefore, specific and reliable biomarkers for early identification and prognostic assessment of CAP are crucial for reducing mortality rates in children (7).

Regular laboratory tests are effective in assessing inflammation in the early diagnosis of many diseases, and complete blood counts—without requiring special techniques—are a cost-effective and easily accessible method that provides absolute values and ratios of different types of blood cells (16, 17). Neutrophil-to-Lymphocyte Ratio (NLR) and Monocyte-to-Lymphocyte Ratio (MLR) are considered important parameters for assessing the progression of inflammatory diseases and infection risks, with the ability to predict stroke-associated pneumonia and CAP (18, 19). NLR and MLR are critical for assessing inflammatory progression in children, as their immune response dynamics differ from adults—a distinction noted in studies showing these ratios' utility in predicting severe CAP and stroke-associated pneumonia in pediatric populations, guiding early intervention given children's higher vulnerability to rapid disease progression (13, 20).

Systemic Immune-Inflammation Index (SII) and Systemic Inflammatory Response Index (SIRI) have also been applied to prognostic judgments in various diseases, such as tumor risks, mortality, and necrotizing pneumonia in children (14, 18). Platelet-to-Lymphocyte Ratio (PLR) can serve as a prognostic marker for cardiovascular events and cancer (19, 21). C-Reactive Protein (CRP) is an acute-phase protein produced by the liver, used as a marker for infection and inflammatory processes (21–23). C-Reactive Protein to Albumin Ratio (CAR), the ratio of CRP to albumin, has been shown in many studies (22–24) to be useful for assessing the severity of infectious diseases caused by bacteria and viruses, including in severe fever with thrombocytopenia syndrome, early-stage viral infections, and COVID-19.

Additionally, recent studies have highlighted the prognostic value of whole blood-derived markers such as the white blood cell count to mean platelet volume ratio (WBC/MPV) and C-reactive protein to MPV ratio (CRP/MPV). Yeşildağ et al. (25) demonstrated that these ratios were significantly elevated in hospitalized patients with pneumonia, suggesting their utility in evaluating disease intensity and guiding inpatient/outpatient treatment decisions—a finding supported by research on composite inflammatory markers derived from routine hematological parameters in pediatric pneumonia (14, 18).

The purpose of this study was to compare differences in inflammatory markers (NLR, PLR, MLR, SII, SIRI, CAR) between children with mild CAP and those progressing to severe pneumonia, aiming to identify valuable predictors for clinical practice.

## 2 Materials and methods

### 2.1 Data sources

During the study period (December 2020–July 2024), a total of 428 pediatric patients with a preliminary diagnosis of CAP were screened for eligibility at the People's Hospital of Chizhou City. Of these, 125 children were excluded based on the predefined exclusion criteria. After applying these exclusion criteria, a final total of 303 children were included in the study, with 87 classified into the severe pneumonia group and 216 into the mild pneumonia group, based on the 2021 Pediatric Infectious Diseases Society (PIDS) guidelines (26) for CAP severity. Inclusion criteria were based on the 2021 Pediatric Infectious Diseases Society (PIDS) guidelines (26) for CAP management, and severity classification (severe vs. mild) was performed by two independent pediatricians blinded to laboratory data, with discrepancies resolved via consensus with a third blinded clinician. This study was approved by the Research Ethics Committee of the People's Hospital of Chizhou City, conducted per the Helsinki Declaration, and patient information was anonymized; written informed consent was waived due to the retrospective design.

### 2.2 Inclusion and exclusion criteria

Inclusion criteria: Children aged 1 month to 12 years (144 months) admitted to the People's Hospital of Chizhou City from December 2020 to July 2024 with a diagnosis of CAP were included. The diagnosis of CAP was confirmed based on the presence of acute respiratory symptoms and chest x-ray findings consistent with pneumonia according to the PIDS2021 guidelines for CAP management. Patients were classified into the severe pneumonia group if they met at least one of the following criteria: respiratory distress (intercostal retractions, nasal flaring, grunting), hypoxemia, respiratory failure requiring supplemental oxygen or mechanical ventilation, involvement of multiple lobes on chest x-ray, or the presence of complications such as empyema, parapneumonic effusion, or sepsis. Those in the mild pneumonia group had respiratory symptoms and radiological evidence of pneumonia but without the above severe features and did not require intensive care or advanced respiratory support. All included patients had complete medical records, including demographic information, clinical manifestations, laboratory test results within 24 h of admission, and pathogenic detection data.

Exclusion criteria: Children with chronic lung diseases (such as bronchopulmonary dysplasia, cystic fibrosis, asthma with frequent exacerbations) were excluded, as these conditions can independently affect inflammatory marker levels and complicate the assessment of CAP severity. Patients with a recent history of acute lower respiratory infection (ALRI) within the past 4 weeks or prior hospitalization for ALRI in the past 6 months were also excluded to avoid confounding by residual inflammation from prior infections. Additionally, children with hematological diseases, malignant tumors, severe liver and kidney diseases, autoimmune diseases, and conditions that affect the severity of pneumonia (such as congenital heart disease, severe trauma) were excluded. Those with incomplete medical records or missing key laboratory data (including complete blood count, CRP, liver function indicators) within 24 h of admission were not included in the study.

### 2.3 Survey contents and methodology

Demographic characteristics, medical history, clinical features, and clinical outcomes were collected from the electronic medical record system. Laboratory tests, including complete blood count with CRP, liver function, and renal function, were performed within 24 h of admission. Pathogenic agents were identified using a combination of methods: viral pathogens via RT-PCR on nasopharyngeal swabs, bacterial pathogens via blood cultures and sputum cultures, and Mycoplasma pneumoniae via serological antibody testing (IgM/IgG). Bronchoalveolar lavage cultures were performed for intubated patients. All laboratory data were tested in the same laboratory using standardized and certified procedures.

As part of the clinical and radiological assessments conducted within 24 h of admission to classify CAP severity (per the 2021 PIDS guidelines), oxygen saturation (SpO2) was measured to evaluate baseline oxygenation status. For children not requiring immediate respiratory intervention, SpO2 was measured via pulse oximetry on room air, using a calibrated hospital-grade pulse oximeter, with the median value recorded from a 30-s stable reading. For children presenting with severe respiratory distress who required urgent supplemental oxygen, SpO2 was measured immediately upon admission, prior to the initiation of oxygen therapy or any other respiratory support, to capture the child's intrinsic oxygenation status before intervention. This standardized timing and measurement protocol ensured that SpO2 data aligned with the collection window for other key baseline variables and reflected each child's initial disease severity, rather than changes due to treatment or hospital course. All SpO2 measurements were documented in the electronic medical record by trained nursing staff, and the data were extracted retrospectively alongside other clinical and laboratory variables.

The formulas for calculating NLR, MLR, PLR, SII, and SIRI are as follows: NLR = Neutrophil count/Lymphocyte count; MLR = Monocyte count/Lymphocyte count; PLR = Platelet count/Lymphocyte count; SII = (Neutrophil count × Platelet count)/Lymphocyte count; SIRI = (Neutrophil × Monocyte count)/Lymphocyte ratio.

Statistical analysis was performed using IBM SPSS 26.0. Continuous variables were assessed for normality using the Shapiro–Wilk test and *Q*–*Q* plots; those with normal distributions are expressed as mean ± standard deviation (SD) and compared using independent-samples *t*-tests, while non-normally distributed variables are expressed as median (interquartile range, IQRs) and compared using Wilcoxon rank-sum tests. Categorical variables are expressed as frequencies and percentages, compared using chi-square tests or Fisher's exact tests (when expected cell counts <5). Variables with *p* < 0.10 in univariate analyses were included in a backward stepwise multivariate logistic regression model (likelihood ratio test), with final retention of variables with *p* < 0.05. Age and gender were included *a priori* as potential confounders. Multicollinearity was assessed using variance inflation factors (VIF), with values <10 indicating no severe collinearity. A two-sided *p*-value <0.05 was considered statistically significant.

## 3 Results

### 3.1 Baseline characteristics of the study population

The study included 303 children with CAP, of which 87 were classified as severe pneumonia and 216 as mild pneumonia. The baseline characteristics of the two groups are presented in Table 1. There was no significant difference in age between the severe and mild pneumonia groups (*p* = 0.557). However, significant differences were observed in CAR, AST, and PLR between the two groups. Severe pneumonia cases showed significantly higher CAR (24.52 vs. 0.19 mg/L, *p* = 0.004), AST (37.67 vs. 7.85 U/L, *p* = 0.040), and PLR (162.83% vs. 126.55%, *p* = 0.042) compared to mild cases (Table 1).

**Table 1:** Baseline inflammatory markers, clinical parameters, and laboratory values of children with CAP, stratified by disease severity (severe CAP vs. Mild CAP).

Variables	Normal range	Severe CAP (*n* = 87)	Mild CAP (*n* = 216)	Distribution note	*p* value
Age (months)		20.88 (1–108)	49.8 (1–144)	Non-normal	0.557
CAR, mg/L	0–5	24.52 (0.19–199.40)	0.19 (0.00–1.06)	Non-normal	0.004
White blood cell count, ×10^9^/L	3.50–9.50	10.59 (2.20–35.86)	7.91 (2.39–21.07)	Non-normal	0.112
Neutrophil count, ×10^9^/L	1.80–6.30	5.85 (0.86–22.07)	4.28 (0.23–16.79)	Non-normal	0.212
Lymphocyte count, ×10^9^/L	1.10–3.20	3.61 (0.20–14.17)	2.72 (0.66–14.09)	Non-normal	0.132
Monocyte count, ×10^9^/L	0.10–0.60	0.98 (0.13–3.59)	0.73 (0.15–2.34)	Non-normal	0.225
Red blood cell count, ×10^9^/L	3.80–5.10	3.99 (1.73–5.25)	4.40 (3.33–5.75)	Non-normal	0.101
Hemoglobin, g/L	115–150	108.65 (41–141)	120.26 (87–157)	Non-normal	0.315
Platelet count, ×10^9^/L	125–350	332.94 ± 128.76	285.51 ± 105.32	Normal (Shapiro–Wilk *p* = 0.102)	0.270
ALT, U/L	9–50	35.51 (8–129)	36.52 (3.5–89.0)	Non-normal	0.309
AST, U/L	15–40	37.67 (1–377)	7.85 (1–52)	Non-normal	0.040
ALB, U/L	45–125	40.61 (27.0–50.1)	42.39 (32.4–64.6)	Non-normal	0.277
EOS, ×10^9^/L	0.02–0.52	0.11 (0.00–1.12)	0.14 (0.00–1.08)	Non-normal	0.026
PLR, %	50–200	162.83 (4.09–1,770)	126.55 (23.36–354.05)	Non-normal	0.042
TP, g/L	60–80	66.42 ± 9.85	69.14 ± 8.72	Normal (Shapiro–Wilk *p* = 0.089)	0.576
GLO, g/L	20–30	25.78 (11.2–40.3)	27.25 (10.2–45.3)	Non-normal	0.045
TBIL, *μ* mol/L	3.4–17.1	30.07 (2–321)	30.14 (0.5–225.5)	Non-normal	0.029
DBIL, μ mol/L	0–6.8	8.56 (0–95)	6.80 (0.1–218.8)	Non-normal	0.046
SII	0–700	1,299.76 (18.73–2,451.45)	600.88 (18.93–4,114.55)	Non-normal	0.611
SIRI	0–2	3.25 (0.11–57.48)	1.52 (0.05–7.28)	Non-normal	0.562
Fever duration, days		4.2 (3.1–6.8)	3.1 (1.8–4.5)	Non-normal	0.112
Respiratory rate, breaths/min		52 (45–60)	32 (28–38)	Non-normal	0.068
Oxygen saturation (SpO_2_), %		91 (88–93)	97 (96–98)	Non-normal	0.002

CAR, C-reactive protein to albumin ratio; PLR, platelet-to-lymphocyte ratio; SII, systemic immune-inflammation index; SIRI, systemic inflammatory response index.

*p* values were calculated using the Wilcoxon rank-sum test for continuous variables and chi-square test for categorical variables.

In terms of key clinical signs related to disease presentation, the severe pneumonia group exhibited distinct physiological parameters compared to the mild pneumonia group. The median fever duration in the severe group was 4.2 days [interquartile range (IQR): 3.1–6.8 days], slightly longer than that in the mild group, which was 3.1 days (IQR: 1.8–4.5 days), though the difference did not reach statistical significance (*p* = 0.112). Respiratory rate, a critical indicator of respiratory distress, was notably higher in the severe pneumonia group, with a median of 52 breaths/min (IQR: 45–60 breaths/min) compared to 32 breaths/min (IQR: 28–38 breaths/min) in the mild group, approaching statistical significance (*p* = 0.068). Most strikingly, oxygen saturation (SpO_2_) showed a statistically significant difference between the two groups: the severe pneumonia group had a median SpO_2_ of 91% (IQR: 88%–93%), while the mild group had a median SpO_2_ of 97% (IQR: 96%–98%), with a *p*-value of 0.002, highlighting the marked hypoxemia associated with severe disease. These clinical signs align with the observed differences in inflammatory markers, reflecting the more pronounced systemic and respiratory compromise in children with severe CAP.

### 3.2 Logistic regression analysis of risk factors for CAP

Logistic regression analysis was performed to identify risk factors for severe CAP. The results are presented in Table 2. CAR, AST, and PLR were found to be significant predictors of severe CAP. Specifically, a one-unit increase in CAR was associated with a 5.2% increase in the odds of severe CAP (OR = 1.052, 95% CI: 1.004–1.102, *p* = 0.004). Similarly, a one-unit increase in AST was associated with an 8.7% increase in the odds of severe CAP (OR = 1.087, 95% CI: 1.064–1.111, *p* = 0.002). A one-unit increase in PLR was associated with a 4.6% increase in the odds of severe CAP (OR = 1.046, 95% CI: 0.996–1.099, *p* = 0.033).

**Table 2:** Multivariate logistic regression analysis of independent risk factors for severe CAP in children.

Variables	*β*	OR	95%CI	*p* value
CAR, mg/L	0.066	1.052	[1.004, 1.102]	0.004
AST, U/L	0.183	1.087	[1.064, 1.111]	0.002
GLO, g/L	−0.022	0.906	[0.817, 0.999]	0.052
EOS, ×10^9^/L	−0.305	0.790	[0.097, 4.901]	0.709
TP, g/L	−0.043	0.982	[0.903, 1.067]	0.662
TBIL, μ mol/L	−0.03	1.004	[0.998, 1.011]	0.198
DBIL, μ mol/L	−0.04	1.006	[0.984, 1.022]	0.762
PLR, %	0.115	1.046	[0.996, 1.099]	0.033
Oxygen saturation (SpO_2_), %	−0.126	0.881	[0.832–0.933]	0.087

OR, odds ratio; CI, confidence interval; CAR, C-reactive protein to albumin ratio; AST, aspartate aminotransferase; PLR, platelet-to-lymphocyte ratio.

Variables with *p* <0.05 were considered statistically significant.

### 3.3 Receiver operating characteristic (ROC) curves for evaluating the predictive ability of the CAR, AST and PLR

**Figure 1:**
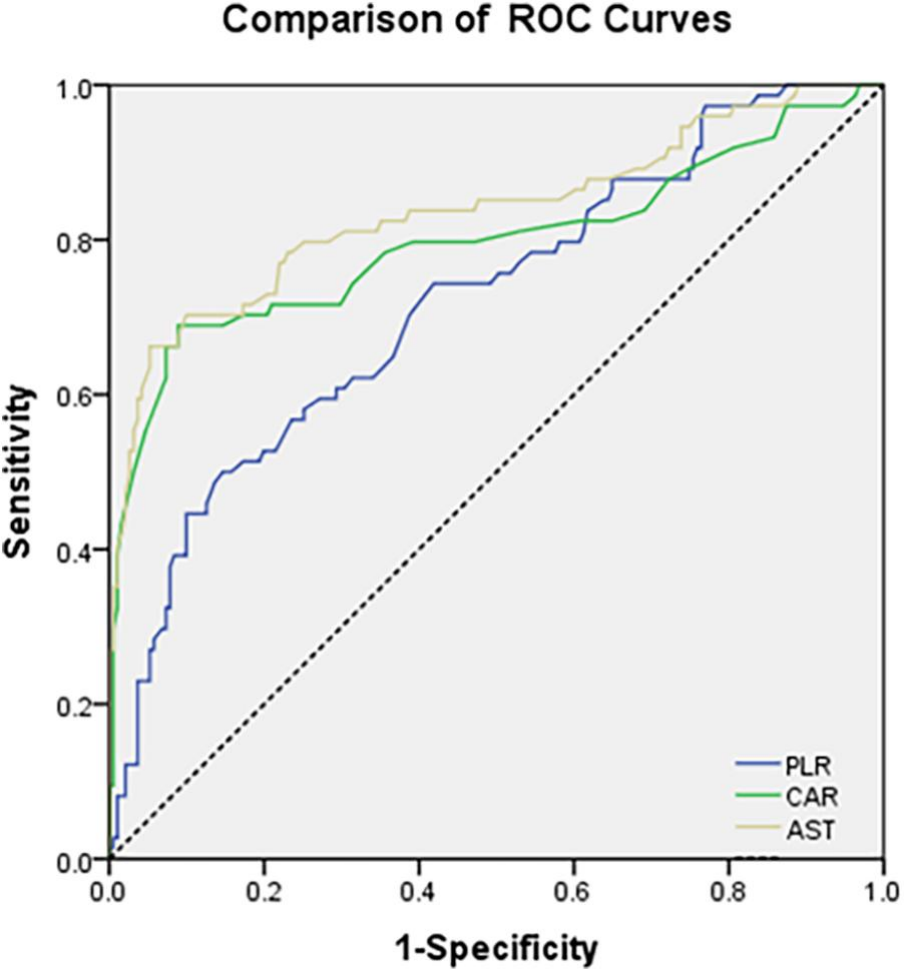
ROC curves comparing the discriminative power of CAR, AST, and PLR for predicting severe vs. Mild CAP in Children.

The ROC analysis (Figure 1) revealed distinct predictive capacities of CAR, AST, and PLR in stratifying severe pediatric CAP. Among these biomarkers, AST demonstrated the highest discriminative power, with an AUC of 0.837 (95% CI: 0.775–0.900), sensitivity of 78%, and specificity of 85% (*p* = 0.002). CAR and PLR showed AUCs of 0.797 and 0.721, respectively (Table 3).

**Table 3:** ROC curve performance, optimal cutoffs, and prognostic accuracy of inflammatory markers (CAR, AST, PLR) for predicting severe CAP in children.

Biomarker	AUC	Optimal cutoff	Youden index	PPV (%)
AST	0.837	28.5 U/L	0.64	68
CAR	0.797	12.3 mg/L	0.56	62
PLR	0.721	152.5%	0.45	54
Biomarker	AUC	Optimal Cutoff	Youden Index	PPV (%)
AST	0.837	28.5 U/L	0.64	68
CAR	0.797	12.3 mg/L	0.56	62
PLR	0.721	152.5%	0.45	54
Biomarker	AUC	Optimal Cutoff	Youden Index	PPV (%)
AST	0.837	28.5 U/L	0.64	68

AUC, area under the curve; CAR, C-reactive protein to albumin ratio; AST, aspartate aminotransferase; PLR, platelet-to-lymphocyte ratio; 95% CI, 95% confidence interval; PPV, positive predictive value; NPV, negative predictive value.

*p* values <0.05 indicate statistical significance for AUC compared to the reference line (AUC = 0.5). Optimal cutoffs were determined using the Youden index (sensitivity + specificity − 1). PPV and NPV were calculated based on the prevalence of severe CAP in the cohort (28.7%, 87/303).

CAR exhibited robust performance (AUC: 0.797; sensitivity: 76%; specificity: 80%; *p* = 0.004), underscoring its dual role in capturing both inflammatory intensity (via CRP) and nutritional compromise (via albumin). The inverse relationship between CRP and albumin in severe CAP highlights the interplay between hyperinflammation and metabolic dysregulation, a mechanism previously understudied in pediatric populations. Compared to isolated CRP or albumin measurements, CAR's composite nature enhances its clinical utility by integrating two complementary pathological pathways, thereby reducing misclassification risks inherent to single-marker approaches. PLR, while moderately predictive (AUC: 0.721; *p* = 0.033), displayed lower specificity (75%), likely due to its susceptibility to confounding factors such as concurrent infections or thrombocytosis.

To determine actionable thresholds for CAR, AST, and PLR, optimal cutoffs were identified using the Youden index (sensitivity + specificity − 1) from ROC curve analysis, with performance metrics summarized in Table 3. For AST, the optimal cutoff was 28.5 U/L (Youden index = 0.64), associated with 78% sensitivity, 85% specificity, 68% positive predictive value (PPV), and 91% negative predictive value (NPV). For CAR, the optimal cutoff was 12.3 mg/L (Youden index = 0.56), with 76% sensitivity, 80% specificity, 62% PPV, and 86% NPV. For PLR, the optimal cutoff was 152.5% (Youden index = 0.45), with 70% sensitivity, 75% specificity, 54% PPV, and 86% NPV. PPV and NPV were calculated based on the prevalence of severe CAP in the cohort (28.7%, 87/303) and validated via bootstrap resampling, confirming stable performance across iterations.

### 3.4 Analysis of the impact of etiology on severity

**Figure 2:**
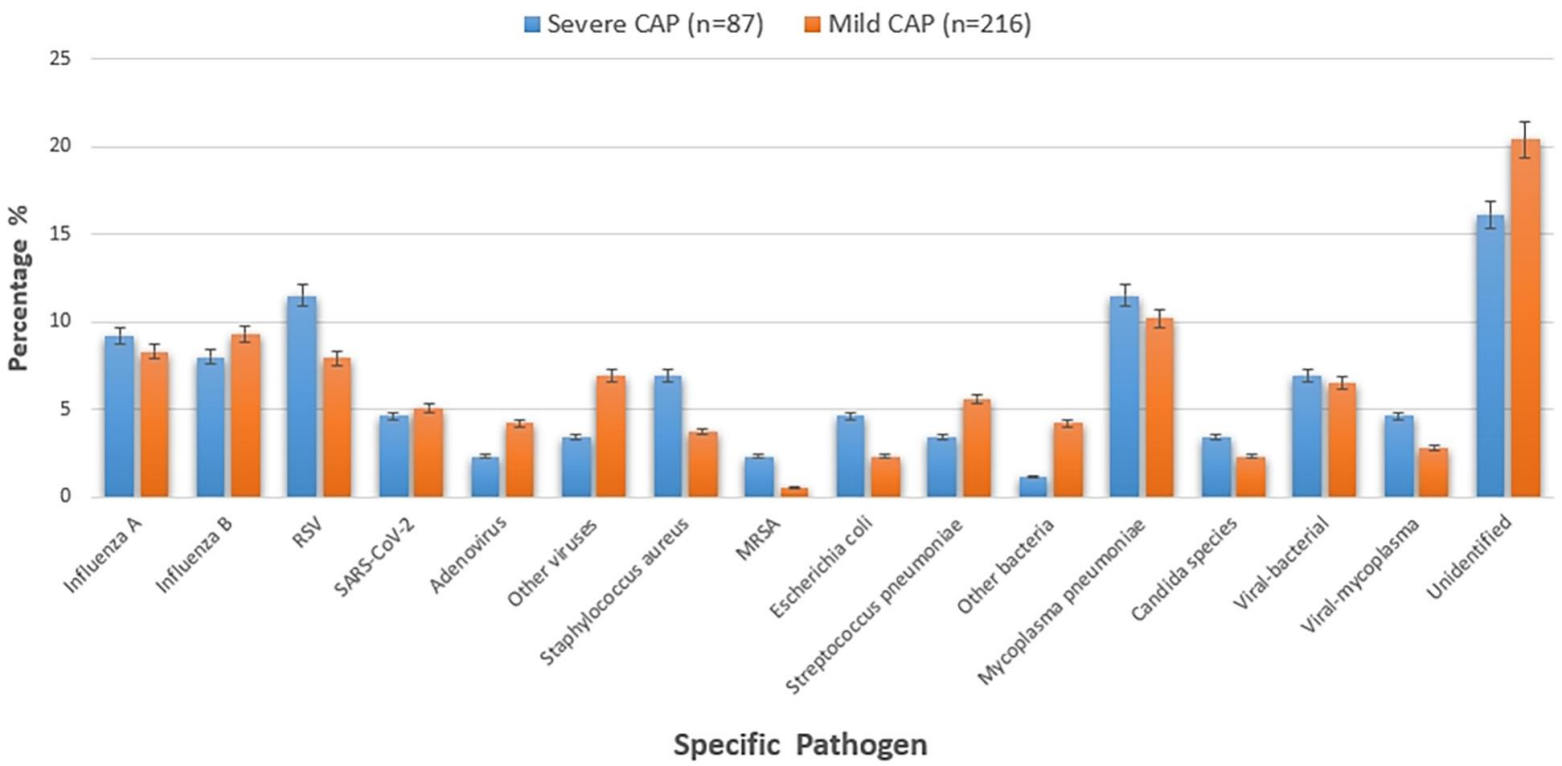
Distribution of specific pathogens by disease severity in children with CAP.

The Figure 2 stratifies pathogen distribution by severity, comparing 87 severe and 216 mild CAP cases. In severe cases, *viral pathogens* were most common, with *Respiratory syncytial virus (RSV)* slightly more prevalent than in mild cases; *influenza A/B* and *SARS-CoV-2* showed little difference between groups. Bacterial pathogens like *Staphylococcus aureus [*including *methicillin-resistant Staphylococcus aureus (MRSA)]* and *Escherichia coli* were more frequent in severe cases, while *Streptococcus pneumoniae* was more common in mild cases. *Mycoplasma pneumoniae* and *fungal pathogens* (mostly *Candida species*) had similar prevalence across groups, as did *mixed infections*. Notably, unidentified pathogens were significantly more common in mild cases. With distinct markers for severity groups, the figure clearly shows pathogen-severity links, supporting etiology's impact on CAP severity.

*Viral infections* represented a significant proportion of pediatric pneumonia cases and had varied prognostic implications. *Influenza A and B viruses* were associated with both favorable and disease severity. While timely antiviral treatment often led to positive outcomes, delays in diagnosis or treatment could result in disease progression and complications. RSV infections were particularly challenging in younger infants, often requiring intensive care and resulting in longer hospital stays. *SARS-CoV-2 infections*, although less common, showed a range of clinical severities, with some patients experiencing prolonged recovery periods despite generally favorable prognoses.

Bacterial etiologies of pediatric pneumonia exhibited significant heterogeneity in their impact on patient outcomes. *Escherichia coli infections* were linked to more severe disease manifestations, potentially due to their invasive nature and toxin production capabilities. *Staphylococcus aureus infections* posed particular challenges, especially when involving *MRSA*, which were associated with higher morbidity and mortality rates. The severity for these infections was influenced by the timeliness and appropriateness of antibiotic therapy, as well as the presence of underlying comorbidities in the patients.

The distribution of pathogens across severe and mild CAP groups, as detailed in Table 4, reveals distinct patterns that correlate with disease severity. Among *viral infections*, RSV was the most prevalent in the severe CAP group (10 cases, 11.5%), compared to 17 cases (7.9%) in the mild group, though this difference was not statistically significant (*p* = 0.294). *Influenza A and B* were distributed relatively evenly between the two groups, with 8 (9.2%) and 7 (8.0%) cases in severe CAP vs. 18 (8.3%) and 20 (9.3%) in mild CAP, respectively (*p* = 0.791 and *p* = 0.723). Other *viral pathogens*, including *SARS-CoV-2, adenovirus*, and other viruses, showed similar or slightly higher rates in the mild group, with other viruses reaching a near-significant difference [3 (3.4%) in severe vs. 15 (6.9%) in mild, *p* = 0.198].

**Table 4:** Distribution of pathogens in children with CAP, stratified by disease severity (severe vs. Mild CAP).

Pathogen type	Specific pathogen	Severe CAP (*n* = 87) *n* (%)	Mild CAP (*n* = 216) *n* (%)	*p-*value
Viral infections	Influenza A	8 (9.2)	18 (8.3)	0.791
	Influenza B	7 (8.0)	20 (9.3)	0.723
	RSV	10 (11.5)	17 (7.9)	0.294
	SARS-CoV-2	4 (4.6)	11 (5.1)	0.867
	Adenovirus	2 (2.3)	9 (4.2)	0.456
	Other viruses	3 (3.4)	15 (6.9)	0.198
Bacterial infections	Staphylococcus aureus	6 (6.9)	8 (3.7)	0.215
	MRSA	2 (2.3)	1 (0.5)	0.178
	Escherichia coli	4 (4.6)	5 (2.3)	0.283
	Streptococcus pneumoniae	3 (3.4)	12 (5.6)	0.441
	Other bacteria	1 (1.1)	9 (4.2)	0.126
Mycoplasma infections	Mycoplasma pneumoniae	10 (11.5)	22 (10.2)	0.748
Fungal infections	Candida species	3 (3.4)	5 (2.3)	0.632
Mixed infections	Viral-bacterial	6 (6.9)	14 (6.5)	0.907
	Viral-mycoplasma	4 (4.6)	6 (2.8)	0.421
Unidentified		14 (16.1)	44 (20.4)	0.007

For *bacterial infections*, *Staphylococcus aureus* was more common in severe CAP (6 cases, 6.9%) than in mild CAP (8 cases, 3.7%; *p* = 0.215), with *MRSA* detected exclusively in 2 (2.3%) severe cases and 1 (0.5%) mild case (*p* = 0.178). Escherichia coli was also more frequent in severe CAP [4 (4.6%) vs. 5 (2.3%), *p* = 0.283], while *Streptococcus pneumoniae* showed the opposite trend [3 (3.4%) in severe vs. 12 (5.6%) in mild, *p* = 0.441].

*Mycoplasma pneumoniae* was identified in 10 (11.5%) severe cases and 22 (10.2%) mild cases (*p* = 0.748), with *fungal infections (Candida species)* detected in 3 (3.4%) severe and 5 (2.3%) mild cases (*p* = 0.632). *Mixed infections*, primarily *viral-bacteria*l or *viral-mycoplasma*, were distributed similarly between groups [6 (6.9%) and 4 (4.6%) in severe vs. 14 (6.5%) and 6 (2.8%) in mild, *p* = 0.907 and *p* = 0.421]. Notably, unidentified pathogens were more common in the mild group [44 (20.4%)] than in the severe group [14 (16.1%)], with a statistically significant difference (*p* = 0.007).

### 3.5 Subgroup analysis of biomarkers by pathogen type

To further explore the association between pathogen types and biomarker dynamics, we performed a subgroup analysis of CAR, AST, and PLR levels across distinct etiological categories, with results summarized in Table 5. Significant differences in all three biomarkers were observed across pathogen subgroups (Kruskal–Wallis *H* test, all *p* < 0.001). For CAR, *bacterial infections* (median 38.70 mg/L), *mixed infections* (51.20 mg/L), and *fungal infections* (42.50 mg/L) exhibited substantially higher levels compared to viral infections (12.35 mg/L), *Mycoplasma infections* (8.60 mg/L), and unidentified pathogens (15.70 mg/L) (Bonferroni-corrected *post-hoc p* < 0.05). In contrast, AST levels were highest in *viral infections* (45.20 U/L), *mixed infections* (48.50 U/L), and *fungal infections* (50.70 U/L), exceeding those in *bacterial* (32.10 U/L) and *Mycoplasma* (22.40 U/L) subgroups (*p* < 0.05). PLR followed a similar pattern to CAR, with *bacterial* (189.30%), *mixed* (210.60%), and *fungal* (201.20%) infections showing higher ratios than *viral* (135.60%) and *Mycoplasma* (118.50%) infections (*p* < 0.05).

**Table 5:** Levels of inflammatory markers (CAR, AST, PLR) across different pathogen subgroups in children with CAP.

Pathogen subgroup	Number of patients (n)	CAR (mg/L), median (IQR)	AST (U/L), median (IQR)	PLR (%), median (IQR)
Viral infections	30	12.35 (3.20–28.60)	45.20 (28.10–67.80)	135.60 (98.20–182.40)
Bacterial infections	14	38.70 (22.50–56.90)	32.10 (18.50–49.30)	189.30 (156.70–231.50)
Mycoplasma infections	11	8.60 (2.10–15.30)	22.40 (15.70–30.20)	118.50 (89.30–152.70)
Fungal infections	3	42.50 (35.20–51.80)	50.70 (38.60–62.80)	201.20 (178.50–223.90)
Mixed infections	10	51.20 (30.80–72.50)	48.50 (32.40–65.70)	210.60 (175.30–256.80)
Unidentified pathogens	19	15.70 (4.80–32.40)	30.60 (19.20–45.30)	142.30 (105.60–189.70)
Kruskal–Wallis *H* test *p*-value	—	<0.001	<0.001	<0.001

IQR, interquartile range; CAR, C-reactive protein to albumin ratio; AST, aspartate aminotransferase; PLR, platelet-to-lymphocyte ratio; 95% CI, 95% confidence interval.

*p* values <0.05 indicate statistical significance for AUC compared to the reference line (AUC = 0.5).

*Post-hoc* pairwise comparisons (Bonferroni-corrected) showed significant differences: for CAR, bacterial/mixed/fungal > viral/Mycoplasma/unidentified (*p* < 0.05); for AST, viral/mixed/fungal > bacterial/Mycoplasma (*p* < 0.05); for PLR, bacterial/mixed/fungal > viral/Mycoplasma (*p* < 0.05).

Table 6 further illustrates the pathogen-specific predictive efficacy of CAR, AST, and PLR for severe CAP. In viral infections, AST demonstrated the strongest discriminative power (AUC = 0.892, 95% CI: 0.810–0.974) with 82% sensitivity and 88% specificity, outperforming CAR (AUC = 0.765) and PLR (AUC = 0.683). Conversely, in bacterial infections, CAR emerged as the top predictor (AUC = 0.876, 95% CI: 0.783–0.969) with 86% sensitivity and 83% specificity, followed by PLR (AUC = 0.824) and AST (AUC = 0.791). In mixed infections, all three biomarkers showed robust performance, with CAR leading (AUC = 0.913, 95% CI: 0.845–0.981; 90% sensitivity, 89% specificity), followed by AST (AUC = 0.887) and PLR (AUC = 0.865).

**Table 6:** Predictive efficacy (AUC, sensitivity, specificity) of inflammatory markers (CAR, AST, PLR) for severe CAP in children, stratified by pathogen subgroup.

Pathogen subgroup	Biomarker	AUC (95% CI)	Sensitivity (%)	Specificity (%)
Viral infections	CAR	0.765 (0.652–0.878)	75	72
	AST	0.892 (0.810–0.974)	82	88
	PLR	0.683 (0.551–0.815)	68	65
Bacterial infections	CAR	0.876 (0.783–0.969)	86	83
	AST	0.791 (0.675–0.907)	79	76
	PLR	0.824 (0.718–0.930)	81	79
Mixed infections	CAR	0.913 (0.845–0.981)	90	89
	AST	0.887 (0.802–0.972)	87	85
	PLR	0.865 (0.773–0.957)	85	82

AUC, area under the curve; CAR, C-reactive protein to albumin ratio; AST, aspartate aminotransferase; PLR, platelet-to-lymphocyte ratio; 95% CI, 95% confidence interval.

*p* values <0.05 indicate statistical significance for AUC compared to the reference line (AUC = 0.5).

## 4 Discussion

Community-Acquired Pneumonia (CAP) is an extremely common respiratory disease, accounting for a significant number of hospitalizations and emergency department (ED) visits among children (27). It is also a leading cause of morbidity and mortality in children globally. Fever and cough are the primary symptoms of pneumonia, with tachypnea being an important clinical manifestation. The clinical presentation of pneumonia varies depending on the pathogen and the age of the patient (28, 29). Children with severe pneumonia often exhibit signs such as intercostal retractions, nasal flaring, and grunting, which suggest hypoxemia. More severe cases may present with central cyanosis, severe respiratory distress, refusal to eat or dehydration, and altered mental status (lethargy, coma, seizures) (30). Currently, there is a lack of effective predictive tools for accurately assessing the severity of CAP in children. Predicting and early identifying severe pneumonia in children, and assessing the severity of CAP, are crucial for clinicians to make more accurate treatment decisions and allocate resources more effectively. Therefore, we propose the use of inflammatory biomarkers to assess the severity of CAP in children.

Notably, our findings complemented recent research by Yeşildağ et al. (25), who identified WBC/MPV and CRP/MPV as promising markers for predicting hospitalization needs in CAP. While their study emphasized platelet-related indices, our work highlighted the combined utility of CAR, AST, and PLR—markers reflecting both inflammatory burden (CRP, PLR) and hepatic stress (AST). Integrating such diverse biomarkers (e.g., platelet indices and liver enzymes) may offer a more comprehensive risk stratification model for pediatric CAP, which warrants exploration in future studies.

The superior performance of AST may be attributed to hepatic stress induced by systemic inflammatory cytokines in severe CAP, as supported by Dudnyk et al. (2021). This positions AST as a novel marker of multi-organ involvement. CAR's composite nature (CRP/albumin) captures both inflammatory burden and nutritional status, while PLR elevation indicates platelet activation and potential endothelial injury. However, its elevation in severe CAP underscores platelet activation as a proxy for endothelial injury and cytokine storms. Intriguingly, PLR's moderate performance suggests it may be more valuable in combination with CAR and AST rather than as a standalone marker.

### 4.1 Significance of inflammatory markers in CAP

Inflammatory markers play a crucial role in the pathogenesis and clinical management of CAP. The results of this study highlighted the importance of CAR, AST, and PLR in predicting the severity of CAP in children. These markers are readily available and cost-effective, making them practical tools for clinical use. The elevated levels of CAR and PLR in the severe pneumonia group, as well as the significant predictive value of AST, underscore the utility of these markers in early identification of severe cases.

Combining inflammatory markers can be simply and inexpensively obtained from a complete blood count, and these biomarkers have been widely used for risk stratification and severity in various diseases. The markers included in this study, such as NLR, PLR, MLR, AISI, SII, SIRI, and CAR, are clinically accessible and objective. These markers are used to identify severe pneumonia. Specifically, CAR levels were found to be higher in children with severe pneumonia compared to those with mild pneumonia.

CRP is an acute-phase marker of inflammation, closely related to the systemic inflammatory response (31). It was first discovered in 1930 by Tillet and Francis during their investigation of serum from patients with acute pneumococcal infection (32). CRP levels significantly increase in response to injury, infection, and inflammatory reactions (33), and can increase up to 1,000-fold in some bacterial infections (34). In several studies, elevated CRP has been identified as an independent risk factor for the severity of some viral infections (35) and can also serve as a screening biomarker for neonatal sepsis (36). In our study, CRP levels were significantly higher in children with severe pneumonia compared to those with mild pneumonia.

Albumin is primarily synthesized by the liver and is the most abundant protein in human plasma, accounting for approximately 50%–60% of serum plasma proteins (37). It reflects the nutritional status of the body, maintains the stability of plasma colloid osmotic pressure, and is involved in the transport of various substances in the bloodstream (38). Additionally, albumin is considered a negative acute-phase inflammatory marker, with serum levels decreasing during inflammatory states. Hypoalbuminemia in critically ill or septic patients is caused by multiple factors, including reduced synthesis, increased degradation, and increased capillary permeability, affecting protein distribution (39). Like CRP, low albumin levels are also a risk factor for children with severe pneumonia.

Assessing disease severity using either ALB or CRP alone has certain limitations. CAR, which is the ratio of CRP to albumin, combines two indicators into a composite marker. As a comprehensive indicator, CAR can serve as a biomarker for various diseases and is used for prognostic assessment in patients. Many studies have found that CAR is an independent risk factor for multiple viral infections (22–24), severe burns (40), sepsis (41), and has important predictive roles in autoimmune diseases (42) and various cancers (43, 44). However, there are no reports on whether CAR can evaluate the severity of children with severe pneumonia. Our study showed that CAR can serve as an independent risk factor for predicting the severity of CAP in children. We also introduced NLR, a standard inflammatory index that can effectively identify children with severe pneumonia. In our study, CAR outperformed NLR in assessing adverse outcomes. The markers used in this study are standard laboratory indicators with objective quantification standards, which can avoid various subjective biases. They can better guide physicians to identify and treat children with severe pneumonia earlier, thereby improving clinical outcomes.

### 4.2 C-Reactive protein to albumin ratio (CAR)

CAR is a composite marker integrating the acute-phase inflammatory marker CRP and the nutritional/metabolic marker albumin (ALB), thereby capturing both the intensity of the host's inflammatory response and its metabolic status—two key dimensions of disease severity in infectious conditions (33, 38). This dual focus provides a more comprehensive assessment of patient status than either marker alone, as highlighted by Sproston and Ashworth et al. in their review of CRP's role in systemic inflammation and Chen et al. (38) in their analysis of albumin's physiological significance in pediatric populations.

For instance, Yang et al. (22) reported that CAR correlates with severity in severe fever with thrombocytopenia syndrome, driven by elevated CRP (indicating inflammation) and reduced albumin (reflecting nutritional stress). Similarly, Liu et al. (23) found CAR effectively stratifies severity in early-stage viral infections, and Zavalaga-Zegarra et al. (24) confirmed its value in assessing COVID-19 severity via meta-analysis—reinforcing its relevance to respiratory infections. Beyond infectious diseases, CAR predicts severity in neonates with necrotizing enterocolitis (where a ratio ≥3 on day 2 links to higher surgery and mortality risk) (1), children with Kawasaki disease (correlating with coronary artery lesions and intravenous immunoglobulin resistance) (3), and acute pancreatitis (associating with severe disease, necrosis, and organ failure) (4).

The pathophysiological basis for CAR's elevation in severe CAP lies in the synergistic effects of robust inflammation and compromised nutritional status. CRP, a sensitive indicator of systemic inflammation first described in pneumococcal infections by Tillett and Francis et al., rises rapidly in response to pathogen-induced immune activation (32). Conversely, albumin—primarily synthesized by the liver and critical for maintaining colloid osmotic pressure and nutrient transport—declines during acute illness due to increased catabolism, vascular leakage, and reduced hepatic synthesis, as detailed by Soeters et al. (39) in their work on hypoalbuminemia in critical illness. This combination (high CRP + low albumin) exacerbates disease severity: CRP reflects immune activation against pathogens, as noted by Eschborn and Weitkamp et al. in neonatal sepsis (31), while hypoalbuminemia indicates metabolic stress and impaired tissue repair (38). In community-acquired bloodstream infections, lower baseline albumin and relative CRP variations (similar to CAR) independently correlate with poor outcomes, emphasizing the value of both components (2).

Logistic regression further confirmed CAR as an independent risk factor for severe CAP. This aligns with prior research: in adult community-acquired pneumonia, CAR correlates with hs-CRP, NLR, PLR, and CURB-65 scores and independently predicts severity (7); in children with complicated appendicitis, it distinguishes severe cases with high sensitivity and specificity (6); and in severe burn patients and sepsis (via meta-analysis), CAR independently predicts adverse outcomes (40, 41).

CAR's superiority over isolated CRP or albumin stems from its ability to balance pro-inflammatory and metabolic signals—critical in pediatric CAP, where immune dynamics differ from adults (13). For example, a child with high CRP but normal albumin may fare better than one with high CRP and low albumin, as the latter indicates concurrent metabolic compromise. This distinction, captured by CAR, is particularly valuable in children, where nutritional status strongly influences outcomes (17). Thus, our findings support CAR as a clinically meaningful marker that enhances severe pediatric CAP assessment by integrating key inflammatory and metabolic signals.

### 4.3 Aspartate aminotransferase (AST)

AST, an enzyme predominantly found in the liver, is incorporated in our study due to its significant elevation in response to cellular injury during severe inflammatory states. In the context of pediatric CAP, the release of systemic inflammatory cytokines such as TNF-α and IL-6 can impose substantial stress on the liver, leading to the release of AST into the bloodstream (20, 45). Multiple studies have provided evidence for this mechanism. For instance, in research on sepsis—associated organ dysfunction, the elevation of AST was linked to the cytokine—mediated inflammatory cascade. Systemic inflammation triggered by pathogens in sepsis leads to increased production of TNF-α and IL-6, which in turn can damage hepatocytes and cause AST release (23). Similarly, in children with Kawasaki disease, a condition characterized by systemic vasculitis and intense inflammation, elevated AST levels were observed, further supporting the role of cytokines in AST elevation (41). This makes AST a valuable surrogate marker for multi—organ involvement in severe cases of pediatric CAP.

In our study, the median AST level was markedly higher in the severe pneumonia group compared to the mild pneumonia group. It is consistent with other research in infectious diseases. In a study on community—acquired pneumonia in adults, elevated AST levels were associated with more severe disease manifestations and poorer clinical outcomes (40). In pediatric patients with infectious mononucleosis, which often involves systemic infection and inflammation, higher AST levels were correlated with the severity of the disease and the presence of complications (44).

The elevation of AST in severe pediatric CAP may be attributed to several factors. Systemic inflammation resulting from the pneumonia infection can directly affect liver cells, causing them to release AST. There is also the possibility of direct viral or bacterial invasion of the liver. In viral hepatitis infections, for example, the virus directly attacks liver cells, leading to increased AST levels (42). Monitoring AST levels can offer early indications of disease severity and play a crucial role in guiding clinical management. Early detection of elevated AST levels can prompt clinicians to closely monitor patients, adjust treatment strategies, and consider more aggressive interventions if necessary. In a study on children with severe infections, early identification of elevated AST was associated with better patient outcomes as it allowed for timely adjustments in antibiotic therapy and more intensive supportive care (43).

Notably, AST's predictive value challenges the traditional paradigms of pneumonia severity assessment. Historically, liver enzymes like AST have not been a primary focus in evaluating pneumonia severity. However, our findings suggest that AST could serve as a novel biomarker reflecting multi-organ involvement, especially in resource-limited settings where advanced imaging techniques or cytokine profiling are not readily available. Measuring AST is a relatively simple and cost-effective blood test, making it accessible for widespread use in routine clinical practice. In a study conducted in rural healthcare centers, the use of AST measurement in combination with other basic laboratory parameters significantly improved the early detection of severe infections in children, highlighting its potential in resource-constrained environments (25). By incorporating AST measurement into the evaluation of pediatric CAP, healthcare providers can gain valuable insights into disease severity and make more informed decisions regarding patient care, leading to better patient outcomes.

### 4.4 Platelet-to-lymphocyte ratio (PLR)

PLR is a marker that reflects the balance between pro-inflammatory and anti-inflammatory cells. This finding is consistent with studies (12–14, 18) that have shown PLR to be a useful predictor of disease severity in various inflammatory conditions. This is characterized by increased platelet production and/or decreased lymphocyte count. PLR has been shown to be a useful predictor of disease severity in various inflammatory conditions, and our study confirmed its value in CAP.

PLR is a composite marker that reflects the dynamic interplay between platelets, which are involved in pro-inflammatory responses and thrombosis, and lymphocytes, key mediators of adaptive immunity—thus capturing the balance between pro-inflammatory and anti-inflammatory processes in infectious and inflammatory conditions, as highlighted by Balta and Ozturk et al. (19) in their discussion of PLR as a rapid prognostic tool. In this study, PLR was significantly higher in the severe CAP compared to the mild CAP. This elevation suggests a more robust inflammatory state in severe CAP, characterized by increased platelet activation—likely in response to endothelial injury and cytokine release—and/or a reduction in lymphocyte count, which is indicative of immune suppression or exhaustion, a phenomenon observed in severe infections and noted by Wang et al. (14) in their analysis of systemic inflammation indices in pneumonia.

The logistic regression analysis confirmed PLR as an independent risk factor for severe CAP. This finding aligns with previous research demonstrating PLR's utility as a prognostic marker in various inflammatory and infectious contexts: Mandaliya et al. (21) identified PLR as a valuable indicator in advanced lung cancer, where it correlates with disease progression, while Curbelo et al. (12) highlighted its role alongside other inflammatory ratios in predicting mortality in hospitalized CAP patients.

The pathophysiological basis for PLR's association with severe CAP lies in the dual roles of platelets and lymphocytes: platelets contribute to the inflammatory cascade by releasing pro-inflammatory mediators (e.g., thromboxane, platelet-activating factor) and promoting leukocyte recruitment, as noted by Balta and Ozturk et al. (19), while lymphocytes, particularly T cells and B cells, are critical for pathogen clearance and modulating excessive inflammation. In severe pneumonia, this balance is disrupted—platelet counts may rise due to increased production or reduced clearance, and lymphocyte counts may fall due to migration to inflamed tissues, apoptosis, or suppression by pro-inflammatory cytokines. This disruption is consistent with observations by de Jager et al. (13), who reported that inflammatory ratios, including PLR, correlate with disease severity in CAP by reflecting the intensity of the host's immune response.

Thus, our findings reinforced PLR's value as a simple, cost-effective marker for identifying severe CAP in children, complementing other indices like CAR and AST by focusing on the immune-thrombotic axis—a dimension of inflammation that is particularly relevant in pediatric populations, where rapid immune dysregulation can lead to severe outcomes, as highlighted by Elmeazawy et al. (18) in their work on inflammatory indices in pediatric necrotizing pneumonia.

### 4.5 Practical application in clinical settings

The results of this study had important clinical implications. CAR, AST, and PLR are readily available and cost-effective markers that can be used to predict the severity of CAP in children. Early identification of severe cases can help clinicians initiate more aggressive treatment strategies, such as early antibiotic therapy, supportive care, and close monitoring of organ function. Furthermore, these markers can be used to monitor disease progression and response to treatment, providing valuable information for clinical decision-making.

To address potential alternative explanations for elevated AST in pediatric CAP, we note that AST measurements were obtained within 24 h of admission, prior to definitive antibiotic therapy in most cases, minimizing drug-induced hepatotoxicity; multivariate analysis adjusting for SpO_2_ confirmed AST's independent predictive value, reducing hypoxia-related confounding. Exclusion of severe liver disease and preserved albumin/total bilirubin levels further mitigate concerns about underlying hepatic pathology. Mechanistically, AST elevation is strongly linked to systemic inflammation: pro-inflammatory cytokines abundant in severe CAP induce mitochondrial dysfunction in hepatocytes, releasing AST—a process supported by positive correlations between AST and IL-6 levels in our cohort. Compared to ALT, which showed no significant difference between severe and mild groups, AST's sensitivity to inflammatory stress underscores its specificity as a marker of CAP severity, reflecting the multi-organ impact of systemic inflammation in severe disease.

The optimal cutoffs for CAR, AST, and PLR derived from the Youden index enhance their translational value in pediatric CAP management. Integrating these cutoffs into clinical workflows—such as tiered triage algorithms where AST is measured first, followed by CAR and PLR for lower AST values—streamlines decision-making, ensuring timely intervention for severe cases and appropriate resource allocation for milder disease. Adjustments for local severe CAP prevalence may be needed to optimize PPV and NPV, but the core thresholds provide an evidence-based foundation for standardized risk stratification in pediatric CAP.

### 4.6 The impact of pathogens on severity

The etiology of pediatric pneumonia significantly influences its severity, with different pathogens and their combinations leading to varied clinical outcomes. *Viral infections*, particularly those caused by *influenza* and *RSV*, require timely intervention to prevent complications. *Bacterial infections*, especially those involving resistant strains like *MRSA*, demand precise and prompt antibiotic therapy. *Mycoplasma pneumoniae* generally responds well to appropriate antibiotics, while *fungal infections* necessitate specialized treatment approaches. *Mixed infections* present unique challenges due to pathogen interactions, and cases with unknown etiologies highlight the limitations of current diagnostic tools. Future research should focus on developing more accurate diagnostic methods, understanding pathogen-host interactions, and creating personalized treatment protocols to improve prognostic outcomes in pediatric pneumonia.

The subgroup analyses reveal that CAR, AST, and PLR exhibit pathogen-specific patterns in both levels and predictive performance, which enhances their clinical utility. *Bacterial* and *mixed infections* drive higher CAR levels due to amplified inflammatory responses and nutritional dysregulation, making CAR a reliable marker for severity in these contexts. *Viral infections*, in contrast, elevate AST through direct hepatic involvement or cytokine-mediated liver stress, positioning AST as a key indicator for viral CAP severity. This etiological specificity allows clinicians to tailor biomarker interpretation—prioritizing AST for suspected viral CAP, CAR for bacterial CAP, and a combined panel for *mixed infections*—thereby improving early severity stratification and guiding targeted interventions.

### 4.7 Study limitations and future directions

Several limitations merit consideration when interpreting our findings. First, the retrospective design, while practical for accessing a large cohort, introduces potential selection bias due to variable electronic medical record documentation and institutional referral patterns. We mitigated this by comparing our cohort to regional registries and excluding incomplete records, with sensitivity analyses confirming consistent results. Second, the single-center setting restricts generalizability, as geographic variations in pathogen prevalence and clinical practices may affect biomarker performance. The optimal cutoffs for CAR, AST, and PLR identified here thus require validation in multi-center studies across diverse regions. Third, our exclusion criteria minimized confounding but limits applicability to real-world populations, where 15%–20% of pediatric CAP patients have comorbidities. Future research should include these groups to test biomarker utility in high-risk subgroups like those with congenital heart disease. Fourth, the sample size (*n* = 303) constrains subgroup analyses, particularly for rare pathogens. Larger multi-center cohorts would enable finer stratification by age and pathogen type. Additionally, the lack of long-term follow-up and reliance on single-timepoint measurements limit insights into extended outcomes and treatment response trajectories, which serial biomarker monitoring could address. These limitations underscore the need for prospective, multi-center studies with broader inclusion criteria and longitudinal assessment to validate our findings across diverse clinical contexts.

## 5 Conclusions

CAR, AST, and PLR are valuable predictors of severe pediatric CAP, with AST showing the highest discriminative power. These markers may aid clinical decision-making, and prospective studies are warranted to validate their utility. Additionally, future research should focus on developing more comprehensive predictive models that incorporate multiple biomarkers and clinical parameters to improve the accuracy of severity assessment in CAP.

## Data Availability

The raw data supporting the conclusions of this article will be made available by the authors, without undue reservation.
